# Modeling of Trace Metal Migration and Accumulation Processes in a Soil-Wheat System in Lihe Watershed, China

**DOI:** 10.3390/ijerph15112432

**Published:** 2018-11-01

**Authors:** Guijie Tong, shaohua Wu, Yujie Yuan, Fufu Li, Lian Chen, Daohao Yan

**Affiliations:** 1 School of Geographic and Oceanographic Sciences, Nanjing University, 163 Xianlin Road, Nanjing 210023, China; guijietong@163.com (G.T.); yyj554145231@163.com (Y.Y.); zlifufu@163.com (F.L.); yandaohao1989@163.com (D.Y.); 2 Institute of Land and Urban-Rural Development, Zhejiang University of Finance & Economics, Hangzhou 310018, China; 3 Guangzhou Marine Geological Survey, 477 Huanshi East Road, Guangzhou 510075, China; DG1527004@smail.nju.edu.cn

**Keywords:** trace metals, wheat growth, pollution, risk evaluation, spatial analysis

## Abstract

Samples of wheat and soil were collected in the Lihe watershed in East China, the migration and accumulation processes of four common trace metals (Cu, Pb, Cd and Ni) in each part of the wheat plant (root, stem, leaf and grain) were analyzed, and a mechanistic model was proposed to simulate these processes based on wheat growth techniques. Model results show that Cu and Cd migrate more easily with wheat grains, while most Pb and Ni accumulate in roots. Modeling results were shown to be relatively good, with an error of 25.29% in value and 26.38% in fluctuation, and had smaller dispersion degree than actual measurement results. Monte Carlo simulation results also match quite well with actual measurement results, and modeling results are slightly smaller in the simulation of Leaf-Cu, Grain-Cu and Leaf-Ni. Trace metal pollution risk in wheat is evaluated based on this model; our results show that the northwest and northeast parts in the research area are not suitable for growing wheat. In general, this model is relatively accurate, and can evaluate the wheat pollution risk before seeding wheat, providing scientific references for the early selection of wheat safety sowing areas.

## 1. Introduction

With the rapid development of global urbanization and industrialization, increasing amounts of trace metals are being emitted into soils through agricultural, industrialization [1,2], and transportation routes [3,4,5]. Previously, researchers analyzed 1200 rice soil samples in the United States, 63% of which were contaminated with Pb, Cr, Cd, and Cu [6], and 472,000 hm^2^ of farmland in Japan was contaminated with Cd [7]. The arctic is also heavily polluted with trace metals, especially river mouths and ports [8]. In China, 19.4% of the arable lands were contaminated by 2014 [9]. Trace metals have 6 kinds of chemical fractionations in soil, in which water-soluble and exchangeable trace metals are more easily absorbed by crop roots, from where they transfer to edible parts [10,11], and migrate to the human body through food webs to endanger people’s health [12,13], especially children, adult females and those living in the most severely-polluted regions [14,15,16]. Cr is carcinogenic, even at low concentrations [17,18], while Zn and Cu can change the function of the human central nervous system and disrupt the endocrine system [19].

The trace metal accumulation process in crops is complex, and easily impacted by different crop species and growth stages, trace metal species and concentrations, soil properties (pH, SOM, CEC and ORP), microorganisms, and atmospheric environments [20,21,22]. Soil trace metals enter wheat roots by one of two ways: diffusion and plasmid flow (transpiration pulling force) [23]. They are transported into wheat stems by symplast and apoplast pathways [24], and migrate to leaves and grains through xylem. Meanwhile atmospheric trace metals can also enter leaves and grains by metabolic and nonmetabolic pathways. Moreover, trace metals can also be transported from top to bottom in phloem by stems and leaves [25,26]. In the previous studies about trace metal accumulation in crops, the contamination factor (CF) [27], pollution load index (PLI) [28], and the ecological risk index (RI) [29] have often been used to assess soil trace metal pollution, while bioconcentration factor (BCF) is often used to compare crop absorption capacity for different trace metals [30], and the translocation factor (TF) can show trace metal migration ability from crop roots to aboveground parts [31]. Pollutant transport processes in soil-crop systems are complex and difficult to quantify. In some studies, organic pollutant transport models have been proposed and used widely [32], while models about trace metal accumulation are relatively rare. The simulation of trace metal accumulation processes in a soil-crop system can be divided into two types: empirical and mechanistic models. With empirical models, Tang established the correlation model between rice Cd and soil Cd and pH in the field through multiple regression analysis [33]. Cheng analyzed the relationship between trace metals in rice soil and DTPA (diethylene triamine pentaacetic acid) effective trace metals in Zhejiang Province by using the regression equation [34]. Zhao discussed trace metal migration in soil-rice systems of hybrid rice and late japonica rice, and established quantitative relationships between the migration accumulation and biological effectiveness and the trace metal distribution in soil, and soil physical and chemical properties [35]. With mechanistic models, Peng established an urban soil trace metal accumulation model based on mass balance and Monte Carlo methods, and simulated Cd accumulation in plants, and pointed out that Cd was mainly affected by the amount of atmospheric deposition in urban soil [36]. Shi established trace metal migration models in soil-crop systems based on wheat growth processes, and took trace metals in atmospheric precipitation into account; this model was more accurate at modeling Cu, Pb, and Ni [37].

In previous simulations, soil properties and atmosphere deposition were usually considered; however, these simulations ignored the crop growth process, even though it is vital for understanding trace metal migration and accumulation processes [38]. Additionally, previous modeling results were not applied to risk assessment, which is significant to display the practicability of the model. We assume that trace metals migrate into wheat through soil and the atmosphere, and accumulate as wheat grows. We aim to establish a mechanism model to simulate trace metal migration in wheat based on the wheat growth process, and evaluate wheat trace metal pollution in the spatial aspect based on this model.

In our study, wheat growth and nutrient uptake processes are modeled first; then, trace metal migration will be modeled based on this process. In addition, the simulation accuracy will be assessed by comparing actual measurement values with sample simulations and Monte Carlo simulations separately. Finally, Wheat trace metal pollution risk will be shown on spatial distribution maps. This model can reduce much of the complicated field work, and yields relatively accurate simulation results; these results can not only provide scientific reference values for trace metal pollution administration, prevention, and control, but can also help to plan the wheat safety zones.

## 2. Materials and Methods

### 2.1. Research Area

**Figure 1:**
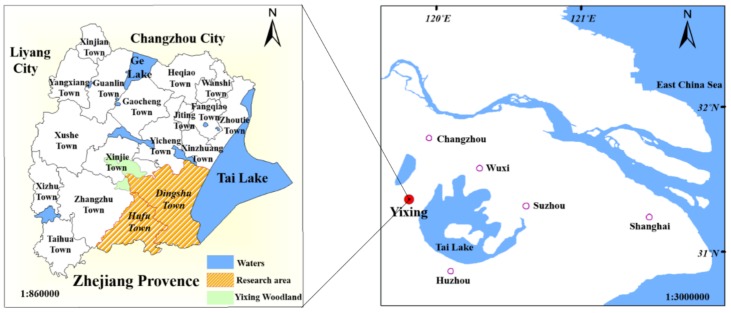
Location of study area.

The research area of this paper is a small watershed of Lihe (including Dingshu Town and Hufu Town) in Yixing City, Jiangsu Province (Figure 1). Yixing is located at 31°07′–31°37′ N, 119°31′–120°03′ E in the Taihu Basin of the Yangtze River Delta. Dingshu Town is located in the Yangtze River Delta economic development zone; it covers an area of 20,500 hm^2^, and is one of the two largest main city zones in Yixing. Hufu Town is located in the junction of Jiangsu, Zhejiang, and Anhui Provinces, and covers an area of 9318 hm^2^. Yixing has a prosperous economy, with an urbanization rate of 64.75%, a per capita GDP of 118,000 yuan, and is usually in the forefront of similar counties throughout China; but, at the same time, many types of industrial plants, including ceramics factories, refractory materials plants, and chemical plants, are densely distributed in research area, and have caused serious trace-metal pollution [39].

### 2.2. Samples Collection and Test

**Figure 2:**
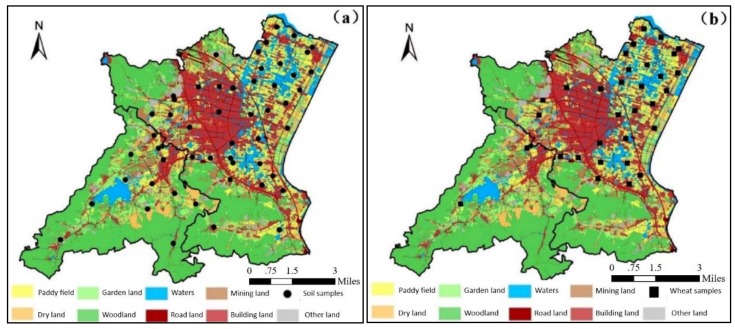
Distribution of (**a**) soil samples, and (**b**) wheat samples.

Wheat planting areas were selected by firstly interpreting remote sensing images; sampling sites were selected by a grid sampling method on wheat planting areas, and we added several sampling sites in heavily polluted areas. We selected 52 sampling sites altogether. Thirty-two wheat samples were mature among the 52 sampling sites; no wheat had been planted or harvested at the 20 remaining points. A three-point-sampling method was used when collecting soil samples; at each site, we collected about 500 g soil samples (0–10 cm deep) and 10 wheat samples, and sealed them in sample bags. Each sample point was recorded in detail and its GPS coordinates were noted. The distributions of samples are shown in Figure 2.

We washed out the soil from the wheat roots and separated wheat samples into roots, stems, leaves, and grains. Later, wheat samples were stoved, weighed, and smashed, before being boiled in mixed acid (HNO_3_-HClO_4_) and measured by ICP-MS. Meanwhile, we used a triacid-melting method (HF-HNO_3_-HClO_4_) to boil and dissolve soil samples, and measured them by ICP-MS (Cd) and ICP-OES (Cu, Pb, Ni). In addition, 10 soil comparison samples and 10 wheat comparison samples were added in our experiment. Meanwhile, we added soil certified reference materials GBW07405 at each interval of 2 soil samples, and added wheat certified reference materials GBW07602 at each interval of 2 wheat samples. The accuracy of tests met the requirements laid out in the Chinese document, *Technical requirements of quality assurance and quality control for detailed investigation of agricultural soil pollution status*. In addition, we also added some blank samples in soil and wheat experiments to correct measurement results.

### 2.3. Wheat Plants Absorb Trace Metals Model

#### 2.3.1. Wheat Growth and Trace Metal Accumulation

(1) $$M_{i}\left(t\right)=\frac{M_{i,\max}}{1+\frac{M_{i,\max}-M_{i,0}}{M_{i,0}}\times e^{-G_{i}\times t}}$$

(2) $$\frac{{\mathrm{dC}}_{i}}{\mathrm{dt}}=r_{S-i}\cdot \left[C_{S}\left(t\right)+C_{t}\left(t\right)\right]\cdot W_{i}+r_{A-i}\cdot \frac{C_{A}\left(t\right)}{M_{i}\left(t\right)}-k_{i}\cdot C_{i}\left(t\right)$$

Wheat absorbs Cu, Pb, Cd, and Ni while growing; at the same time, these trace metals will also produce toxic effects on wheat growth, which are very complex and not considered here. Additionally, the research area is flat, and wheat species are homogenous, so terrain slope and wheat species are also not considered. In Formula (1) [40], M_i_ is the mass of part i in wheat (kg); M_i,max_ and M_i,0_ represent the maximal mass (kg) and initial mass (kg) of part i, respectively; G_i_ is the growth coefficient; t is time (d). In Formula (2), C_i_ is trace metal total contents (mg·kg^−1^); C_s_ is the water soluble concentration (mg·L^−1^); and C_t_ is the exchangeable concentration (mg·L^−1^). In addition, r_s−i_ and r_A−i_ represent the down-up (soil to wheat) and up-down (atmosphere to wheat) absorption rates, respectively; W_i_ is the water content of part i in wheat (L·kg^−1^); C_A_ is the trace metal content in the atmosphere (mg·m^−3^); k_i_ is the trace metal loss rate of part i in wheat (d^−1^); and the loss rate k is the total rate at which trace metals migrate from each part to other parts of the wheat plant [32,40].

#### 2.3.2. Trace Metal Absorption Rate in Wheat

**Figure 3:**
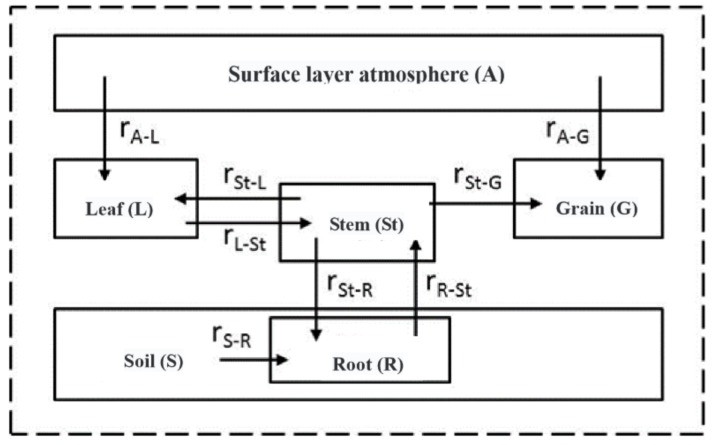
Absorption rate in wheat.

The formula of each part of the plant’s trace metal absorption rate is shown in Table 1, and a trace metals absorption overview is shown in Figure 3. In Formula (3) and (4) [32], r_i−j_ is the absorption rate at which part j absorbs trace metals from part i in wheat; in this process, we only consider down-up and up-down migration rather than diffusion, and this process mainly includes r_R−St_, r_St−L_, r_St−G_, r_L−St_, and r_St−R_. In addition, r_S−R_ is the roots’ absorption rate for soil trace metals, and its formula includes two parts, because trace metals can enter wheat roots by plasmids flow and diffusion separately.

Wheat plants absorb trace metals not only from soil but also from near-surface atmospheric deposition by leaves and grains. As is shown in formulas (5) and (6) [40], r_A−L_ and r_A−G_ represent the trace metal absorption rates from the atmosphere in wheat leaves and grains separately. Their calculations consist of two parts because the trace metals in the atmosphere can enter wheat leaves and grains via metabolic and non-metabolic pathways.

(3) $$r_{i-j}=\frac{Q_{j}}{K_{\mathrm{iw}}\times M_{j}}$$

(4) $$r_{S-R}=\frac{\left(Q+A_{R}\times f_{c}\times D_{R}\right)}{M_{S}\cdot K_{\mathrm{SW}}}$$

(5) $$r_{A-L}=A_{L}\times P_{L}\cdot \left(1-f_{P}\right)+A_{L}\cdot v_{\mathrm{dep}}\cdot f_{p}$$

(6) $$r_{A-G}=A_{G}\times P_{G}\cdot \left(1-f_{P}\right)+A_{G}\cdot v_{\mathrm{dep}}\cdot f_{p}$$

In these formulas, Q_i_ is the flux of part j in wheat (L·d^−1^), K_iw_ is the partition coefficient between part i and water (L·kg^−1^), and M_j_ is the weight of part j (kg). In addition, A_R_, A_L_, and A_G_ represent the surface area of roots, leaves, and grains (m^2^), respectively, Q is the flux in wheat roots (L·d^−1^), f_c_ is a volume conversation parameter (L·m^−3^), D_R_ is the diffusion rate of elements in roots (m·d^−1^), M_s_ is the weight of soil (kg), and K_SW_ is the partitioning coefficient between soil and water (L·kg^−1^). In addition, P_L_ and P_G_ represent the permeability of wheat leaves and grains (m·d^−1^), respectively; f_P_ is the adsorption rate of atmospheric particulate matter (-), and v_dep_ is the deposition rate of atmospheric particulate matter (m·d^−1^).

### 2.4. Parameters

The necessary data for modeling wheat absorption of trace metals include soil, atmosphere, wheat, partitioning coefficient, etc. Among them, the soil trace metal concentration, trace metal deposition flux, trace metal contents of each wheat part, and some other data are derived from experiments. The parameters and data sources are listed in Table 2.

### 2.5. Accuracy Evaluation

There are two ways of evaluating the model’s accuracy: comparing modeling results with actual measured results on value and fluctuation (discrete degrees), and comparing Monte Carlo simulation results (10,000 times) with actual measured results.

#### 2.5.1. Verification of the Modeling Results

In the value aspect, root mean square (RMS) is calculated to compare the model results and measured results, and the value difference rate (V_DR_) is identified based on RMS to show their values’ differences. In the fluctuation aspect, coefficient of variance (CV) is calculated to show the different discrete degrees between modeling and measured results on the whole, and the fluctuation difference rate (F_DR_) is identified to show difference rates in terms of fluctuation.

(7) $$X_{\mathrm{rms}}=\sqrt{\frac{\sum_{i=1}^{N}X_{i}^{2}}{N}}$$

(8) $$V_{\mathrm{DR}}=\frac{\left|X_{\mathrm{rms}\_\mathrm{mod}}-X_{\mathrm{rms}\_\mathrm{mea}}\right|}{X_{\mathrm{rms}\_\mathrm{mea}}}$$

(9) $$\mathrm{CV}=\frac{\mathrm{STD}}{M}$$

(10) $$F_{\mathrm{DR}}=\frac{\left|{\mathrm{CV}}_{\mathrm{mod}}-{\mathrm{CV}}_{\mathrm{mea}}\right|}{{\mathrm{CV}}_{\mathrm{mea}}}$$

In these formulas, X_rms_ is the root mean square (RMS), X_i_ is trace metals contents, V_DR_ represents the accuracy extent on value aspect, X_rms_mod_ is the RMS of modeling value, and X_rms_mea_ is the RMS of measurement value. CV is the coefficient of variance, STD is the standard deviation, M is the arithmetic mean. F_DR_ is the fluctuation difference rate, CV_mod_ is modeling coefficient of variance, while CV_mea_ is measured.

#### 2.5.2. Monte Carlo Verification

Monte Carlo simulation is a way to estimate the probability of something happening by many repeated experiments [44]. Monte Carlo simulation is usually divided into two steps: find targeted variables and determine their distribution characteristics and variation ranges, then generate numerous values of targeted variables, and apply their random values to the target model.

In Monte Carlo simulations, the number of random values determines the accuracy of the simulation. To make the comparison more intuitive, the Monte Carlo simulation’s probability density curves and actual sample measurement results’ frequency distribution histogram will be drawn on the same diagram, and the model’s accuracy will be evaluated by comparing its frequency distributions.

## 3. Results

### 3.1. Wheat Growth Process

**Figure 4:**
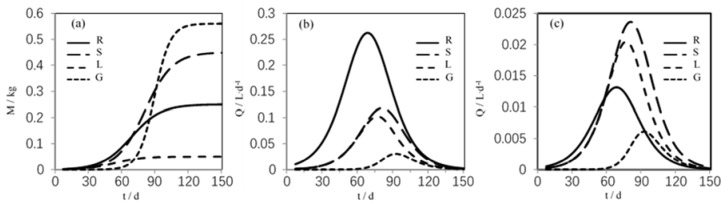
Changes in mass and flux over time in the wheat growth process. (**a**) Mass; (**b**) Flux (down-up); (**c**) Flux (up-down).

Basing on the initial mass M_i,0_, maximum mass M_i,max_, and growth rate G_i_ of each wheat part (Table 3), we can simulate the mass and flux change of each wheat part in the growing process (Figure 4). The flux change considers both xylem transport (down-up) and phloem transport (up-down). It will take approximately one week for the wheat plants to sprout, and then roots, stems, and leaves begin to grow, while grains appear approximately 50 d after sowing.

In general, grains and stems grow faster, and roots and leaves grow slowly. At approximately 90 d, the mass of wheat roots, stems, and leaves begin to stabilize, and their growth rates become slow; the grains’ masses begin to stabilize at approximately 110 d. In addition, phloem transport flux is shown to be less than xylem transport flux by comparing each wheat part. In addition, the root transport flux is the biggest in the down-up process, the leaf and stem transport flux is higher in the up-down process, and the grain transport flux is small in both processes.

### 3.2. Trace Metal Accumulation Process

**Figure 5:**
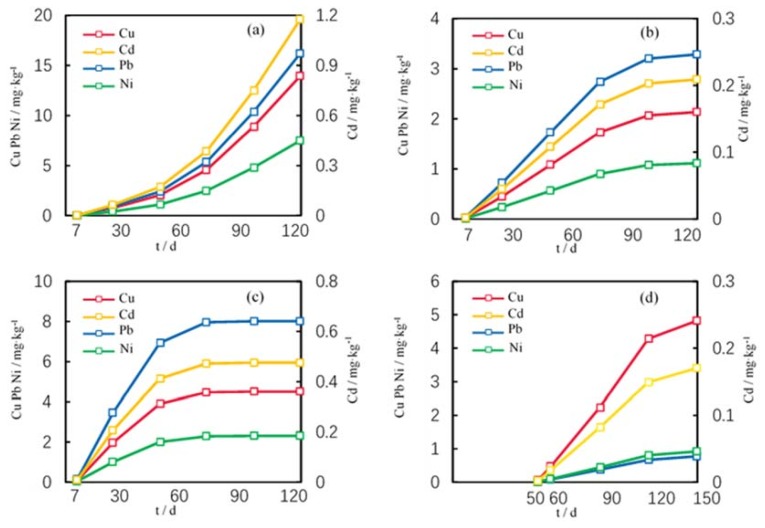
The accumulation process of Cu, Cd, Pb and Ni in wheat parts. (**a**) Root (**b**) Stem (**c**) Leaf (**d**) Grain.

In the wheat growth process, the trace metal accumulation in roots, stems, leaves, and grains exhibits large differences (Figure 5). (1) Roots: Soil trace metals enter wheat roots by diffusion and transpiration pulling. Trace metals in wheat roots accumulate slowly during the initial growing period, but with the growth of wheat, the accumulation rate increases constantly; (2) Stems: Small amounts of trace metals are transported from roots to stems by symplast and apoplast transport, and another portion is derived from the near-surface atmospheric deposition and up-down transport in wheat. In the initial growth period, the levels of trace metals in stems continually increase, and the accumulation rate slows down at approximately 120 d after sowing; then, the trace metals contents gradually become stable and reach a maximum in the ripe period; (3) Leaves: Trace metals in the leaves accumulate rapidly in the initial growth process, and at approximately 90 d after sowing, the trace metal contents become stable and reach the maximum concentration; (4) Grains: Grains appear approximately 50 d after sowing, and accumulate trace metals rapidly in the initial growth period; the accumulation gradually slows down in the late growth period.

The simulation results show that different parts of wheat have different accumulation capacities for trace metals, and all trace metal contents are highest in wheat roots, followed by the leaves, and then by the stems and grains (Table 4). Each part of wheat has different accumulation capacities for different trace metals, and the trace metal contents show an order of Pb, Cu > Ni > Cd in roots, stems, and leaves, and Cu > Ni > Pb > Cd in grains.

In the research area, trace metals concentration in soil (C_soil_) varies with the type of trace metals, and different trace metals have different capacities to transfer from soil to wheat (BCF_sw_ = C_wheat_/C_soil_) or to grains (BCF_sg_ = C_grain_/C_soil_), and accumulate. Cu, Pb, and Ni have higher contents in soil, while Cd migrates more easily to wheat; Cu and Cd accumulate more easily in grains than Pb and Ni (Table 5).

### 3.3. Accuracy Evaluation

#### 3.3.1. Verification of the Modeling Results

**Figure 6:**
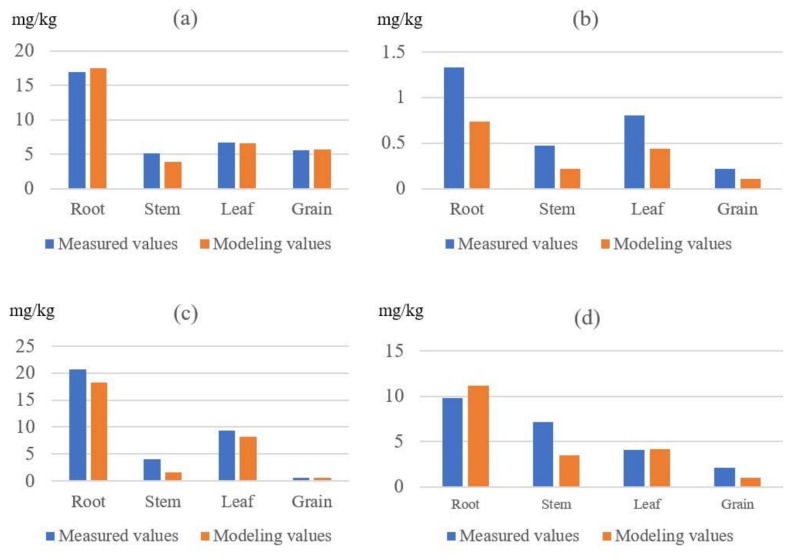
Root mean squares (RMS) of measured values and modeling values, (**a**) the RMS of Cu, (**b**) the RMS of Cu, (**c**) the RMS of Cu, (**d**) the RMS of Cu.

The RMS of actual measurement results and modeling results are shown in Figure 6. Four kinds of trace metals contents all show an order of root > leaf > stem, grain, and modeling results are slightly smaller than measured. Cu and Pb show better simulation results than Cd and Ni.

**Figure 7:**
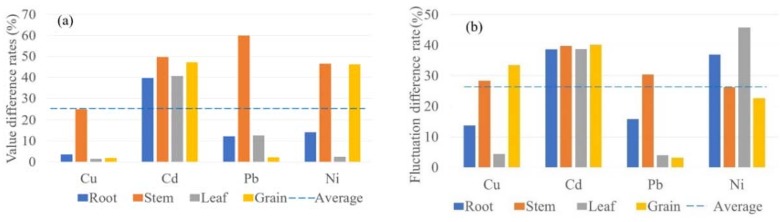
(**a**) Value difference rates (V_DR_) and (**b**) Fluctuation difference rates (F_DR_) of the modeling results.

The value difference rate (V_DR_) was calculated based on RMS, and fluctuation difference rate was calculated based on CV (Figure 7). Modeling results were shown to be relatively good, average value difference rate is 25.29%, average fluctuation difference rate is 26.38%, and modeling results are slightly stable in numerical distribution (CV_mod_ = 0.5, CV_mea_ = 0.63). Cu and Pb are all better simulated both on value and fluctuation, while Cd has relatively poor simulation results.

#### 3.3.2. Monte Carlo Verification

As the number of simulations given above is too low to assess the model’s accuracy accurately, Monte Carlo simulation was used to assess the accuracy.

**Figure 8:**
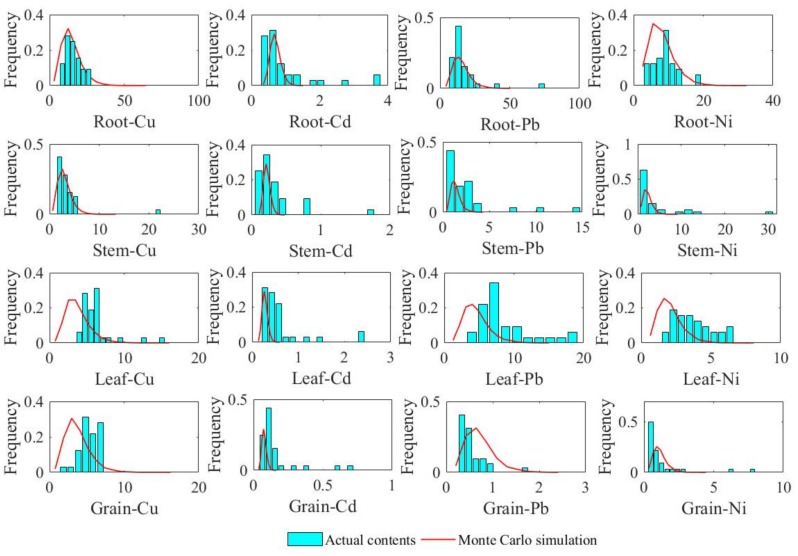
Comparison between Monte Carlo simulation and actual measurement results.

In research area, soil trace metal contents vary from sample to sample, and a large difference between different soil samples exists. Meanwhile, atmosphere trace metal contents have smaller differences, and it is difficult to determine their distribution. Therefore, soil trace metal content was selected to be the test variable to verify the model’s accuracy. The logarithms of four kinds of measured soil trace metals contents were first calculated; their *p* values are all greater than 0.05 (K-S normality test), so they all obeyed normal distribution approximately, i.e., 10,000 random values (assume them x) of soil trace metals contents were generated based on this normal distribution formula. We calculated exp(x) to revert simulative values from normal distribution to previous actual distribution; these reverted values are the X axis in Figure 8. These values were applied to our model, which was run 10,000 times. We then calculated the corresponding trace metals concentrations in wheat (Y axis).

To make the comparison more intuitive, the Monte Carlo simulation and actual measurement results were drawn on the same diagram (Figure 8), where the green bars are the frequency distribution histogram of actual trace metal contents, while the red lines represent probability density curves of Monte Carlo simulation. Overall, the Monte Carlo simulation results match pretty well with the actual contents, and they have similar frequency distributions. Meanwhile, small deviations also exist for the Leaf-Cu, Grain-Cu, and Leaf-Ni; Monte Carlo simulations were slightly smaller than actual contents.

### 3.4. Risk Evaluation of Wheat Trace Metal Pollution

This model is accurate and reliable, and can assess not only the trace metal pollution risk from existing wheat, but also predict the pollution risk in areas where no wheat is grown, based on soil trace metal contents. Evaluation results can assess not only existing wheat grain safety, but also provide scientific advice for the planning of future wheat safety zones.

**Figure 9:**
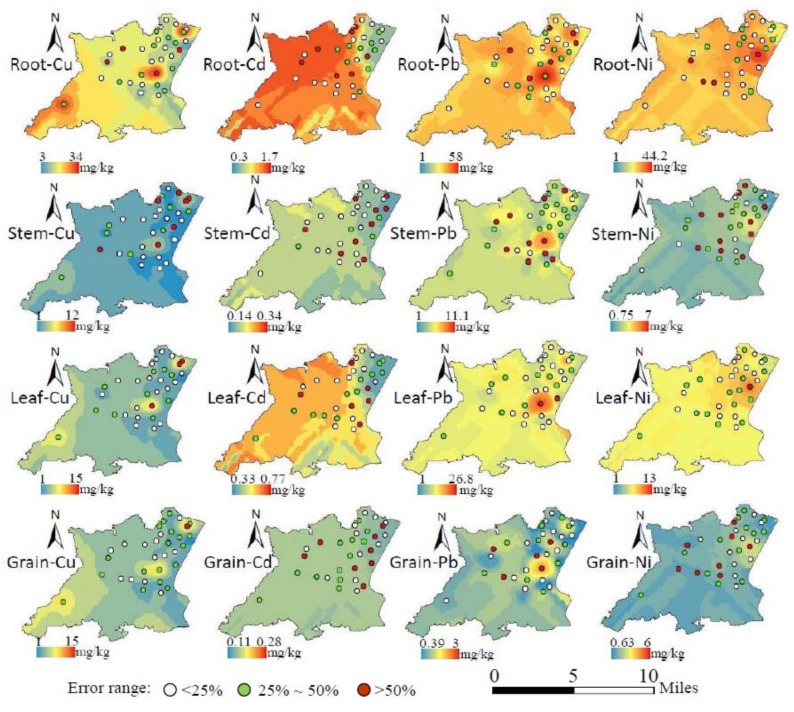
Risk evaluation of trace metals pollution in wheat.

Wheat trace metal contents were simulated based on our model, and a spatial distribution map was draw by kriging interpolation (Figure 9); the semi-variogram and prediction error of each kriging interpolation map is shown in Supplementary Materials . Blue represents lower trace metal concentrations, while red is higher. Thirty-two wheat samples prediction values were extracted from the kriging interpolation map and compared with measured values, and the error ranges (|prediction−measured|/measured) were calculated and divided into three ranges: <25%, 25%–50% and >50%, and expressed in different colors.

In comparing these maps in the horizontal direction, we observed that Cu poses a high pollution risk in the northeast and southwest, Cd’s high pollution risk zone is in the northwest, while Ni and Pb both have higher pollution risks in the northeast. In the vertical direction, their concentrations all follow a sequence of root > leaf > stem, grain. In addition, the simulation of Cu is relatively accurate, while Pb and Ni have several inaccurate points in the central region, and the inaccurate points of Cd are in the eastern region.

## 4. Discussion

Trace metal (Cu, Pb, Cd, and Ni) accumulation processes in soil-wheat systems were modeled accurately in this study. This model is based on the growth process of wheat, and considers trace metal migration processes in all wheat parts (root, stem, leaf, and grain). Modeling results are displayed in space, and can provide scientific guidance for evaluating wheat trace metal pollution, and selecting wheat safety sowing areas.

Modeling results show that different parts of wheat have different trace metal contents and accumulation capacities. Four trace metals contents show an order of root > leaf > stem, grain. The bioconcentration factors from soil to wheat (BCF_sw_) show an order of Cd > Cu > Pb > Ni, but there is a different order from soil to grain (BCF_sg_): Cu > Cd > Ni > Pb, which is consistent with previous studies [45]. Meanwhile, there are small differences of BCF_sg_ in some studies; they show an order of Cd > Cu > Ni > Pb [46,47] or Cd > Cu > Pb > Ni [48]. In general, our simulation results and previous studies all show the order of Cu, Cd > Pb, Ni. Compared with wheat, rice has similar laws, and previous studies have found that trace metal contents show the sequence of paddy soil > root > straw > grain, which is same as our model results of wheat [49]. Cd migrates more easily to rice grains than Pb, Cu, and Ni [50,51]. In addition, wheat is more likely to accumulate trace metals than corn; corn leaves have higher trace metals contents than stems and roots [52], and the BCF of corn grain is Cd > Cu > Pb [47]. In summary, Cd has the strongest migration capacity, and accumulates more easily in grains in these crops, while Pb does not migrate effectively to grains; most of it accumulates in the root. These different migration capacities may be due to internal and external factors. Internal factors: different trace metals chelation, compartmentalization, adsorption and translocation processes are different in soil and crops, they are as components of a complex ‘firewall system’ which acts in limiting trace metal translocation from the root to the shoot, and which reaches different equilibrium positions depending on trace metals external concentration [53]. External factors: field management methods will affect trace metal migration capacity; for example, Cd migrates more easily to wheat grains in organic farms than conventional farms [54], sewage irrigation will improve the translocation of Cu and Ni while decreasing Pb’s migration in wheat [55], and mulching may promote the accumulation of Cd and Pb in vegetables [56].

To evaluate the model’s accuracy, modeling results and Monte Carlo simulation results are compared with actual measurement sample results. Comparison results show that the modeling results are relatively good, with an error of 25.29% in value and 26.38% in fluctuation. Modeling results have a smaller dispersion degree than actual measurement results, and the model’s Monte Carlo simulation results match pretty well with the actual measurement results. In general, Cu, Pb, and Ni have better simulation results, and their mean modeling errors (V_DR_) are 7.89%, 21.67%, and 27.28% separately, while Cd’s modeling error is 44.31%. Compared with other trace metals (Cu, Pb, and Ni), the content of Cd is much smaller than those of the other metals, whether in soil or in wheat. In soil, Cd’s content is almost all less than 1 mg/kg, while other trace metals are all present at greater than 10× that amount. That means soil that Cd is more susceptible to exogenous interference. Additionally, Cd-rich irrigation water, and other pollution emissions will all affect the accuracy of the model to a large extent. Therefore, more relevant factors should be considered in further improvement of this model, such as the quality of irrigation water and field management methods.

Wheat trace metal pollution risk was evaluated based on this model. Meanwhile, spatial distribution maps were created by kriging interpolation. Modeling results show that Cu poses a high pollution risk in the northeast and southwest near lakes and some mining areas. Cd’s high pollution risk zone is in the northwest, while Ni and Pb both pose a higher pollution risk in the northeast near building lands. Building and mining areas make up a relatively high proportion (17.3%) of the study area, and hundreds of ceramic enterprises exist there. Trace metal pollution is often more serious around these areas, and the pollution caused by industrial emissions (water, gas and slags) needs to be given more attention. In addition, the simulation of Cu is relatively accurate, while Cd has relatively poor simulation, and many inaccurate points are distributed in the eastern region, because it has many lakes. Meanwhile, wheat irrigation methods are different from the west region, and soil Cd content is relatively low in this region. Therefore, Cd is more susceptible to other factors (irrigation, field management) than other trace metals in this region.

Some soil trace metals transport inefficiently into grains, but cluster easily in roots. For example, Pb and Ni are abundant in soil and roots, but only a small portion can be absorbed by wheat grains. This indicates that wheat may be a better choice for phytoremediation of soil Pb and Ni than some other native plants [57], and that wheat grains may still be suitable for human consumption even when soil trace metal contents are high. It also shows that leaf trace metal contents are relatively high, and that more attention should be paid when preparing fresh plant food for herbivores. This model is relatively accurate in the simulation of Cu, Pb, and Ni, and we can judge whether the area is suitable for planting wheat before sowing this basis, and provide scientific reference for selecting sowing areas. We will also be able to choose crop species to be sown and the management to be adopted after the improvement of the model.

The research area has a flat terrain, and many properties are approximately uniform, so this model doesn’t contain many factors. But, in fact, many the literature has pointed out that parent materials, soil properties (pH, SOM, etc.), terrain slop, plant species, temperature, the quality of irrigation water, and field management methods all have complex influences for plants absorbing trace metals. In further research, natural and human factors should be considered in the model. In nature, we should find another wheat planting area where the natural environment has larger variability, the relevance between each influential factor and wheat trace metals contents should be calculated, and high-relevant factors should be added to this model as tentative variables. In terms of human factors, population, field management, polluting enterprises, and traffic pollution should also be considered to make this model more accurate and more widely-applicable.

## 5. Conclusions

A mechanistic model was established to simulate trace metal migration in wheat. Cu, Pb, and Ni have the best simulation results in this model, and Cu and Cd migrate more easily to grains, while Pb and Ni tend to accumulate in roots.

We assumed that trace metals were absorbed constantly during the growth of wheat, and a model was proposed based on the wheat growth process. The source of trace metals and the transport process in wheat are all considered, and modeling results are displayed intuitively in space. Modeling results are relatively good, with an error of 25.29% in value and 26.38% in fluctuation. The model’s Monte Carlo simulation results also match pretty well with the actual measurement results. Cu, Pb, and Ni have better simulation results, while Cd has a relatively poor simulation in the eastern region, because soil Cd content is relatively low in this region, and is more susceptible to other factors (irrigation, field management) than other trace metals.

Model results show that four trace metal contents show the order of root > leaf > stem, grain in wheat, while the bioconcentration factors from soil to grain (BCF_sg_) show an order of Cu > Cd > Ni > Pb. Model results are consistent with previous studies [45], despite slight differences due to wheat species or study area, but all the BCF orders were Cu, Cd > Pb, Ni [46,47,48]. We found that Pb and Ni are abundant in soil and roots, and only a small portion can be absorbed by wheat grains. Therefore, it may be that this wheat variety can provide good phytoremediation for Pb and Ni. Meanwhile, we can judge whether the area is suitable for planting wheat before sowing based on this model.

In further study, more natural and human factors should be considered. Plant species should be carefully considered and taken as a factor in our model. In the human aspect, the field management method has an important effect on trace metal accumulation processes, and should be considered in further studies. We should know where is safe for sowing, which crop is planted, and which management method should be adopted based on our model. In addition, crop straw is directly used as animal feedstuff, so we should also assess the risk of trace metal pollution in straw based on this model, and provide a “safe straw area” for local farmers.

## Figures and Tables

**Table 1:** Absorption rate formulas for trace metals in each part of wheat.

Item	Root (R)	Stem (St)	Leaf (L)	Grain (G)
r_S−i_	r_S−R_	r_S−R_ r_R−St_	r_S−R_ r_R−St_ r_St−L_	r_S−R_ r_R−St_ r_St−G_
r_A−i_	r_A−L_ r_L−St_ r_St−R_	r_A−L_ r_L−St_	r_A−L_	r_A−G_

**Table 2:** Main parameters and sources.

Data Type	Parameter	Source
**Soil data**	Soil weight M_S_	This study
	Soil trace metal contents C_s_, C_t_	This study
**Atmosphere data**	Trace metals contents in the atmosphere C_A_	This study
	Adsorption rate of atmospheric particles f_p_	Rein, etc., 2011 [40]
	Deposition velocity of particles v_dep_	Rein, etc., 2011 [40]
**Crop data**	The initial mass of wheat each part M_i,0_	This study
	The maximum mass of wheat each part M_i,max_	This study
	The water content of wheat each part W_i_	This study
	The surface area of wheat each part A_i_	This study
	The growth coefficient of wheat each part G_i_	Rein, etc., 2011 [40]
**Partitioning coefficient**	Soil-water K_Sw_	Fantke, etc., 2011 [32]; Hung, 1997 [41]
	Atmosphere-water K_Aw_	Rein, etc., 2011 [40]
	Root-water K_Rw_	Fantke, etc., 2011 [32]
	Stem-water K_Stw_	Fantke, etc., 2011 [32]
	Leaf-water K_Lw_	Fantke, etc., 2011 [32]
	Grain-water K_Gw_	Fantke, etc., 2011 [32]
	Flux Q	Rein, etc., 2011 [40]; Hung, 1997 [41]
**Other data**	Volume conversion factor f_c_	Fantke, etc., 2011 [32]
	Diffusion rate D_R_	Verma, 2006 [42]
	The permeability of leaf and grain P_L_, P_G_	Trapp, etc., 2007 [43]

**Table 3:** Initial mass, maximum mass and growth rate in each wheat part.

Item	M_i,0_ (kg)	M_i,max_ (kg)	G_i_
**Root (R)**	0.0025	0.25	0.0075
**Stem (St)**	0.00125	0.45	0.08
**Leaf (L)**	0.00125	0.05	0.08
**Grain (G)**	0.0000056	0.56	0.14

**Table 4:** The modeling results of trace metals contents in wheat/mg·kg^−1^.

Element	Cu	Cd	Pb	Ni
**Root** (R)	16.25 ± 6.76	0.72 ± 0.14	16.26 ± 8.42	9.14 ± 6.56
**Stem** (St)	3.55 ± 1.48	0.21 ± 0.04	1.44 ± 0.7	2.84 ± 2.04
**Leaf** (L)	6.07 ± 2.52	0.43 ± 0.08	7.31 ± 3.79	3.40 ± 2.44
**Grain** (G)	5.25 ± 2.18	0.1 ± 0.02	0.54 ± 0.28	0.82 ± 0.59

**Table 5:** Wheat absorption (BCF_sw_) and grain absorption (BCF_sg_) capacities for different trace metals.

Element	Cu	Cd	Pb	Ni
**C_soil_**/mg·kg^−1^	22.1	0.6	36.3	28.9
**BCF_sw_**	1.41	2.43	0.7	0.56
**BCF_sg_**	0.24	0.17	0.01	0.03

## Supplementary Materials

The semi-variogram and prediction error of each kriging interpolation map (Figure 9) are available online at http://www.mdpi.com/1660-4601/15/11/2432/s1.
