# Supplementary material for: Modeling of Trace Metal Migration and Accumulation Processes in a Soil-Wheat System in Lihe Watershed, China
Source: Int J Environ Res Public Health. 2018 Nov 1;15(11):2432. doi: 10.3390/ijerph15112432 (PMC6266972; doi:10.3390/ijerph15112432)
Supplement: Supplementary file 1 [file ijerph-15-02432-s001.pdf]

International Journal of
Environmental Research and Public Health

The semi-variogram and prediction error of each kriging interpolation map (Figure 9) are as follows:

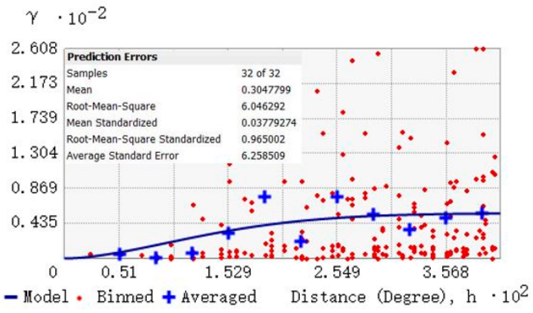

Figure S1. The semi-variogram of Root-Cu.

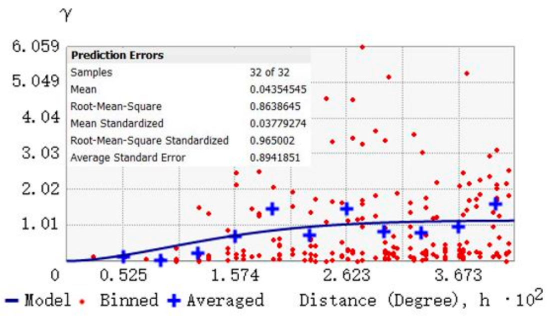

Figure S2. The semi-variogram of Stem-Cu.

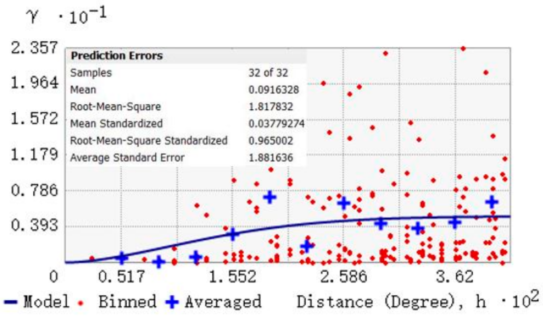

Figure S3. The semi-variogram of Leaf-Cu.

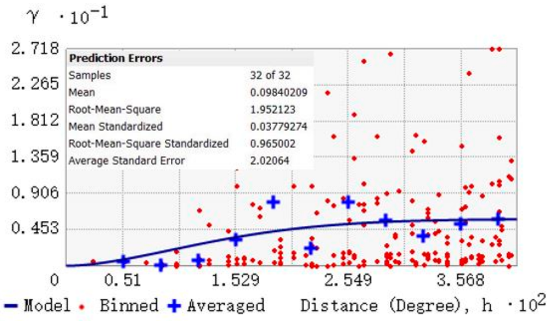

Figure S4. The semi-variogram of Grain-Cu.

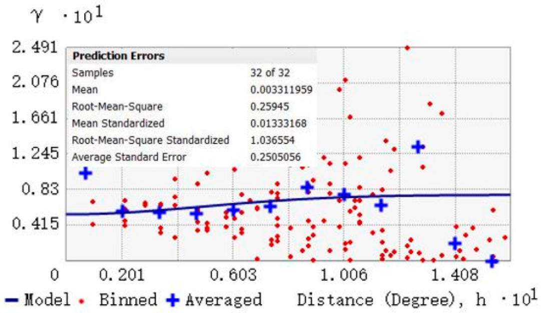

Figure S5. The semi-variogram of Root-Cd.

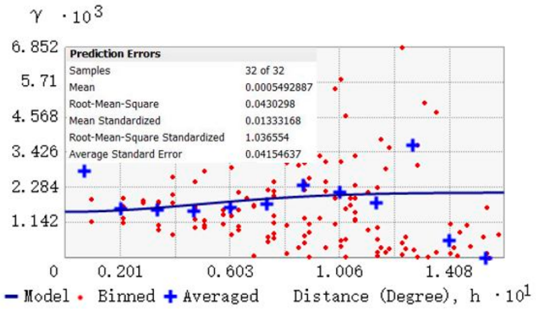

Figure S6. The semi-variogram of Stem-Cd.

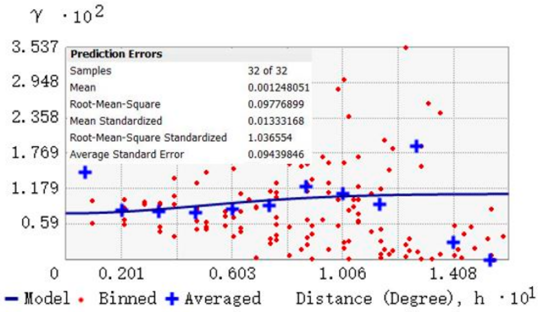

Figure S7. The semi-variogram of Leaf-Cd.

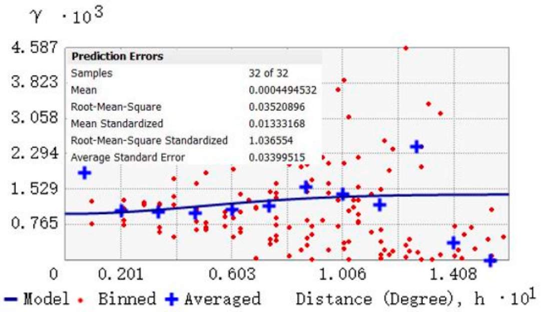

Figure S8. The semi-variogram of Grain-Cd.

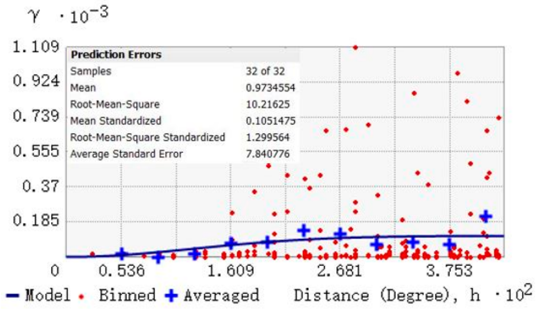

Figure S9. The semi-variogram of Root-Pb.

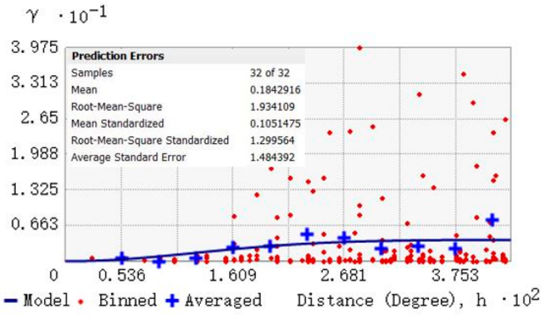

Figure S10. The semi-variogram of Stem-Pb.

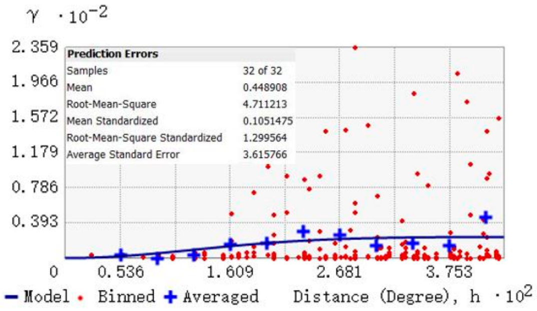

Figure S11. The semi-variogram of Leaf-Pb.

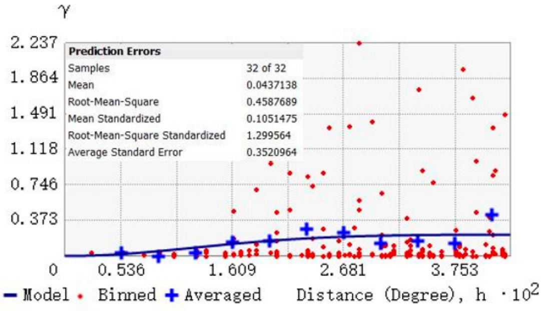

Figure S12. The semi-variogram of Grain-Pb.

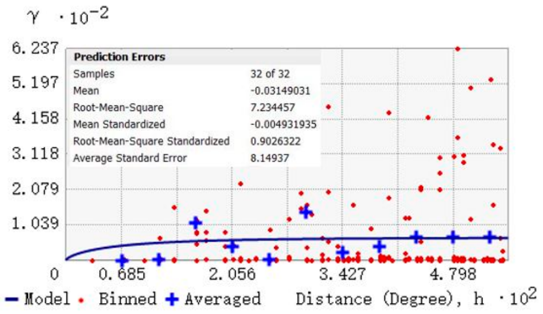

Figure S13. The semi-variogram of Root-Ni.

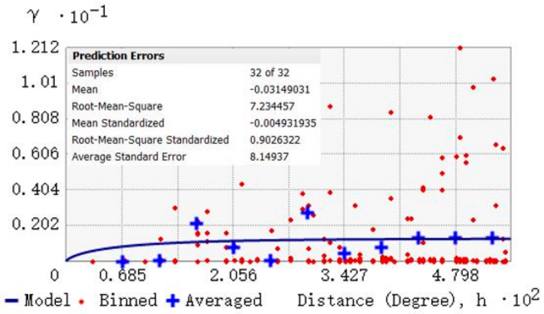

Figure S14. The semi-variogram of Stem-Ni.

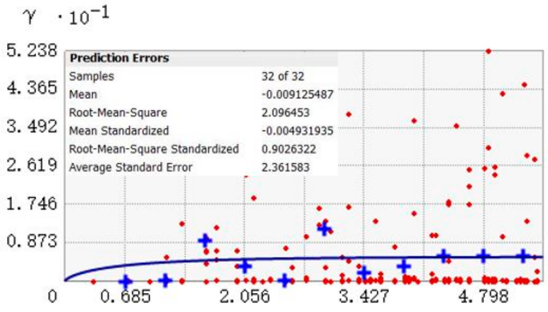

Figure S15. The semi-variogram of Leaf-Ni.

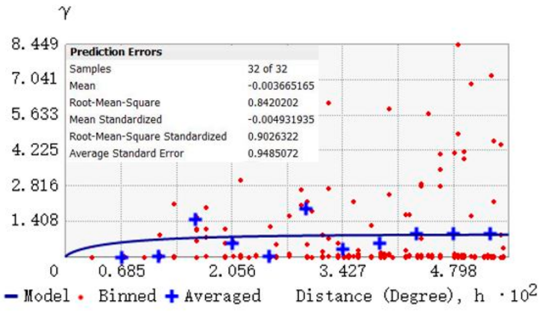

Figure S16. The semi-variogram of Grain-Ni.
